# Loss of ARNT Limits Improvement in Physiological Performance Following Aerobic Exercise in Aging

**DOI:** 10.1093/geroni/igaa057.1582

**Published:** 2020-12-16

**Authors:** Yori Endo, Yuteng Zhang, Bin Li, Michael MacArthur, Indranil Sinha

**Affiliations:** 1 Harvard Medical School, Boston, Massachusetts, United States; 2 Harvard T.H. Chan School of Public Health, Boston, Massachusetts, United States

## Abstract

Hypoxia signaling is essential for angiogenesis and metabolic regulation during exercise. Our previous study has demonstrated an age-related loss of ARNT resulting in limited muscle regeneration. To explore the role of hypoxia signaling in physiological performance in relation to aging, we generated a mouse model with skeletal muscle-specific knockout of ARNT (ARNT mKO). ARNT mKO and ARNT WT mice were subjected to a sedentary activity or treadmill running exercise regime at an increasing speed of 8-12 m/min for 40 minutes, three times weekly over the course of 8 weeks. ARNT levels was 3-fold lower in old mice compared to young. The exercised WT mice exhibited 52% greater increase over the sedentary group in exercise endurance as measured by the maximum running distance (490.92±154.28 vs 237.76±135.19m, p＜0.01). In contrast, ARNT mKO mice did not benefit from exercise (231.85±198.61 vs 167.27±136.56m, p=0.41). The maximum running speed was severely restricted in the trained ARNT mKO mice versus WT (16±1.63 m/min vs 26.67±2.45 m/min, p＜0.001). Cross-sectional area of myofibers increased significantly following exercise in WT mice (2270 vs 2960 □m2, p=0,015) indicating muscle hypertrophic response, while no change was observed in the ARNT mKO group (2101 vs 2378□m2, p=0,21). Further, exercise increased femoral artery blood flow by 41% in ARNT WT mice, but not in ARNT mKO mice (898.96±52.33 vs 802.86±48.43, p=0.20). These data suggest that ARNT is essential for physiological response to exercise

